# On Applicability of Network Coding Technique for 6LoWPAN-based Sensor Networks

**DOI:** 10.3390/s18061718

**Published:** 2018-05-26

**Authors:** Marek Amanowicz, Jaroslaw Krygier

**Affiliations:** 1 NASK—National Research Institute, Kolska 12, Warsaw 01-045, Poland; marek.amanowicz@nask.pl; 2 Faculty of Electronics, Military University of Technology, Urbanowicza 2, Warsaw 00-908, Poland

**Keywords:** network coding, wireless sensor networks, 6LoWPAN, delay-tolerant sensor networks, Internet of Things

## Abstract

In this paper, the applicability of the network coding technique in 6LoWPAN-based sensor multihop networks is examined. The 6LoWPAN is one of the standards proposed for the Internet of Things architecture. Thus, we can expect the significant growth of traffic in such networks, which can lead to overload and decrease in the sensor network lifetime. The authors propose the inter-session network coding mechanism that can be implemented in resource-limited sensor motes. The solution reduces the overall traffic in the network, and in consequence, the energy consumption is decreased. Used procedures take into account deep header compressions of the native 6LoWPAN packets and the hop-by-hop changes of the header structure. Applied simplifications reduce signaling traffic that is typically occurring in network coding deployments, keeping the solution usefulness for the wireless sensor networks with limited resources. The authors validate the proposed procedures in terms of end-to-end packet delay, packet loss ratio, traffic in the air, total energy consumption, and network lifetime. The solution has been tested in a real wireless sensor network. The results confirm the efficiency of the proposed technique, mostly in delay-tolerant sensor networks.

## 1. Introduction

In recent years, wireless networks have become present in almost every area of our lives. Growing demand for mobile services provision enforces careful bandwidth allocation to each wireless network, which, as a consequence, may limit its capacity. Moreover, new mobile applications generate higher traffic streams, which can overload the network. Besides, the Internet is a continuously growing network, covering Wireless Sensor Networks (WSNs) and machine to machine communications. This idea refers to the so-called Internet of Things (IoT), where things can communicate while using heterogeneous networks. Most things require an autonomous power supply (mostly small battery), thus they use low power consuming wireless techniques. Traffic streams in such networks can be sent in multihop transmissions, where packets are forwarded hop by hop to a destination. In many cases, the WSNs use IEEE 802.15.4-based sensor motes with a small data rate. However, traffic that is generated by IoT applications can easily overload such networks. Many solutions have appeared in recent years, which can be applied in these networks to increase traffic handling efficiency, and consequently, to decrease the overall traffic volume [1,2,3,4,5]. One of them is the Network Coding (NC) technique that was introduced by Ahlswede et al., in [6] and was then used in many solutions, discussed for example in [7,8,9,10,11,12,13,14,15,16,17,18,19,20,21,22,23]. It is defined as a “technique, where a node (nodes) receives information from all the input links, encodes, and sends information to all the output links” [6]. This technique can be used in telecommunication networks for many purposes, e.g., to reduce the network load, to increase the packets delivery ratio, to improve the reliability or the security of communication, to improve energy efficiency, etc.

The main goal of the NC implementation in WSNs is to increase the network lifetime through decreasing the network load. The reduction of wireless transmissions is crucial for energy saving in sensors motes. The majority of sensors’ energy is used by Radio Frequency (RF) transceivers. For example, the CC2420 single-chip used in Tmote Sky motes consumes maximum 23 mA in receive mode and 21 mA in transmit mode (at 0 dB), as compared with 2.4 mA that is used by microcontroller in operating mode and 21 µA in standby mode [24]. In order to achieve this goal, the inter-session NC mechanism (called as NC6LoWPAN), which is located between Medium Access Control (MAC) and adaptation (6LoWPAN) layers, is proposed. It can be implemented in WSNs with different MAC protocols without any additional modifications of the protocol stack. Additionally, this enables the implementation of the NC6LoWPAN in the networks with resource (battery, memory) limitation. The inter-session coding works in enforced coding opportunity, i.e., packets are fractionally delayed in sensor nodes to perform coding. Thus, the network throughput (and consequently energy efficiency) can be improved, even if this leads to increase in the end-to-end packet delay. Moreover, it occurs that slight delay in packets delivery can be acceptable for many services in so called Delay-Tolerant Sensor Networks (DTSN) [25]. Therefore, the network coding technique that is discussed in this paper supplements many solutions for non-6LoPWAN networks that have been presented so far [7,8,9,10,11,12,13,14,15,16,17,18,19,20,21,22,23].

The main contribution of this paper as compared to the previous work is:

- the proposal of the relatively simple solution allowing for the encoding of small 6LoWPAN datagrams (fragments) characterized by compressed headers and which are often changed from hop-to-hop,
- the reduction of the number of signaling messages keeping the NC usefulness for the WSNs, and
- the implementation and verification of the NC proposal in a real 6LoWPAN-based testbed.

The NC6LoWPAN mechanism was implemented in Contiki Operating System (OS) [26] and it runs both on the IEEE 802.15.4-based Atmel AVR RAVEN and Tmote Sky sensor motes. The configuration attributes as well as the main parameters of the sensors are described in Section 4. The implementation of the NC6LoWPAN in a real testbed shows a practical usage and verification of the NC technique in an IPv6-based WSN. Most of the papers, in dealing with the NC solutions for WSNs describe the simulation results. 

The remainder of the paper is organized as follows: Section 2 presents prior work related to the NC adoption in wireless ad-hoc networks, Section 3 describes the NC6LoWPAN network coding technique and principles of its implementation in WSN mote, Section 4 depicts the testing methodology and presents the obtained results. Section 5 concludes this paper by summarizing the most relevant results and outlining possible future work.

## 2. Related Work

Most network coding solutions are dedicated for wireless ad-hoc networks; however, other applications are also possible. For instance, networks with overlay P2P services, as presented in [11,12], are a good example of encoding the streams of data that are shared between different positions in the Internet, in order to increase file sharing efficiency. But, due to overhearing, wireless medium gives natural opportunity for network coding. One of the most influential, practical inter-session network coding mechanisms, named COPE, was proposed by Katti et al., in [13]. COPE was elaborated to improve the throughput of IEEE 802.11-based ad-hoc wireless network. The opportunistic unicast packet encoding is performed based on bitwise XOR (Exclusive Or) operations. A similar approach can be found in [14], where also XOR encoding is executed, not in a whole network, but in the so-called capacity region. The NC operations are based on flows instead of individual packets. Both of the solutions ([13,14]) give significant network throughput improvements (from 20 to 80%). A couple of extensions of COPE that offer throughput improvement are presented, for example, in [15,16,17]. Distinctly fewer solutions are devoted to network coding for wireless sensor networks. It should be noticed that sensor motes are characterized by restricted memory resources, which limit the space that is required for efficient network coding operation. In [18], Guo et al., proposed a combined network coding and multipath routing scheme for underwater sensor networks, which is efficient for error recovery and energy consumption. The reliable data dissemination protocol, which uses adaptive network coding to reduce broadcast traffic in sensor networks, is proposed in [19]. This technique is based on the dynamic adjustment of a number of coefficients, and then, the number of packets that can be encoded depending on link density (number of neighboring nodes). Similarly, in [20], network coding is used for the management of available energy during broadcast transmission in a sensor network. It is based on a cross-layer approach that supports the random linear network coding at the network layer, allowing for the cooperation with the MAC layer’s forwarding and scheduling schemes, in order to determine whether and how broadcast packets are encoded. The enhancement of the sensor network lifetime, using duty cycle and network coding, was also discussed in [21,27,28]. Some recent papers are also focused on the increase in the transmission reliability in sensor networks. Amdouni et al., in [22], proposed a broadcast protocol that uses an inter-session network coding scheme, based on the sliding encoding window, which positively influences the efficiency of packet streams transmission. A very interesting solution is presented by Zhu et al., in [23]. To increase a packet delivery ratio in 6LoWPAN-based sensor network, the authors proposed the intra-session network coding to encode the fragments of IPv6 packets that are typically sent in 6LoWPAN-based networks. To decrease an overhead, which is caused by the coding vector that is attached to each encoded packet, 2-byte field of powers of coefficients from Vandermonde matrix are attached, instead of full coefficient vectors. This solution allows for avoiding the retransmissions of frames, and therefore for decreasing overall traffic in a lossy sensor network. 

One of the important issues in sensor networks is a scalability problem. A lot of sensors can be deployed in some region; thus, networks are often organized hierarchically using clustering approach. The NC technique application in cluster-based, error-prone networks is discussed in [29], where the impact of cluster size on approximate decoding performance and data transfer rate is analytically studied. To improve the decoding performance of approximate decoding, a position information matrix (PIM) is used. Presented simulation results confirm that an optimal cluster size can be determined considering the PIM overhead and a target decoding performance. This approach is interesting but should also be analyzed in terms of practical implementation in WSNs. 

The NC has also been proposed recently for routing support in variety of sensor networks. The example solution, named Cooperative and Adaptive Network Coding for Gradient Based Routing (CoAdNC-GBR), is described and discussed in [30]. The CoAdNC-GBR considers the network density as dynamically defined by the average number of neighboring nodes, to perform linear network coding operations on the interest messages. Authors of [30] argue that the cooperation of multiple sinks that were used in the proposed routing mechanism, improves the transmission reliability of links lowers the number of transmissions and the propagation latency and enhances the energy efficiency of the network when comparing to typical gradient-based routing schemes. The NC application for underwater wireless sensor network routing protocol can also be found in [31], where cross-layer interactions are used to mix transport layer packets and the maintenance and correction of paths in the network are operated along with data packets. Simulation results confirm that this solution significantly improves the network performance in terms of energy consumption, end-to-end delay, and packet delivery ratio as compared with other routing protocols. The other proposals for WSNs that support both routing and network lifetime are described in [32,33,34]. 

Most of the solutions that are mentioned above are mainly aimed at an increased packet delivery ratio and an increased reliability of broadcast (multicast) transmission in IEEE 802.11-based networks. The main difference between the network coding schemes, dedicated for sensor network when comparing the solutions for other wireless networks, is maximal simplicity forced by limited resources. Currently, a limited number of network coding-based solutions exist, allowing for us to decrease the unicast traffic in 6LoWPAN-based networks. Bearing in mind that the 6LoWPAN standard is proposed for the IoT architecture, we can expect significant growth of traffic in such networks what can lead to overload and decrease in the network lifetime. In order to increase the WSN lifetime, through decreasing the network load, the authors of this paper propose the NC6LoWPAN inter-session network coding technique for encoding unicast streams of fragments of IPv6 packets. The 6LoWPAN standard assumes that IPv6 packets can be compressed and fragmented, while these operations can change the format of the datagrams from hop-to-hop. Almost all of the similar solutions on the NC, which are presented in the papers, assume that encoded packets should contain some portion of the original packets that are exactly the same as the buffered packets (to ensure decoding). This paper proposes an appropriate NC header that deals with the changing 6LoWPAN headers. The packet buffering mechanism, which is tailored to the available sensor motes resources, is used to increase coding opportunity, and in consequence, to decrease the overall traffic in 6LoWPAN-based WSNs, and in consequence to minimize energy that is consumed by the sensor motes. 

## 3. Network Coding for 6LoWPAN-Based Wireless Sensor Networks

### 3.1. Linear Network Coding Technique

**Figure 1:**
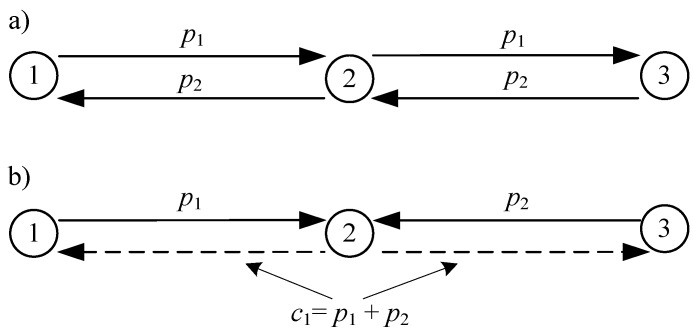
Example of information exchange without (**a**) and with inter-session network coding (**b**).

The idea of network coding was introduced in 2000 by Ahlswede, Cai, Li and Yeung in [6], where the authors found that a node cannot only receive and forward packets, but also encode the received packets (map them on other packets), and send the encoded packets to nodes that are able to decode the original packets. It was argued that, while using network coding, the bottleneck problem can be solved in a traditional wireline network. The packets encoding mechanism is, in most cases, based on linear mathematical operations on at least two original (native) packets. A linear network coding theory was promulgated by Yeung and Cai in [7] and Koetter and Medard in [8]. It assumes that a portion of data (packets, segments, symbols...), which is generated or retransmitted by a node (encoder), can be linearly combined (encoded), giving the vector of the encoded packets. The encoded packets are then sent over the network together with a vector of coefficients used during encoding and decoding. The decoder (or decoders) can decode the original packets after receiving some portion of the encoded packets, while using relatively simple mathematical calculations. The selection of the encoding technique is based on the applied criteria of network performance. Generally, two types of network coding processes exist [35]. Intra-session network coding is typically used to increase the packet delivery ratio in networks with lossy links [22], while inter-session network coding is considered to grow the throughput of the network [14]. The basic concept of the latter technique that is suitable for implementation in resource limited WSNs can be explained using a simple network, as presented in Figure 1. Two nodes (1 and 3) exchange the packets *p*_1_ and *p*_2_. Unfortunately, both packets cannot reach their destinations directly. Figure 1a shows that the packets *p*_1_ and *p*_2_ are forwarded by node 2. Typically, node 2 can transmit the packets sequentially (in consecutive slots or after gaining access to the wireless medium).

In contrary, Figure 1b shows that node 2 performs a linear coding operation, producing one encoded packet *c*_1_ *= p*_1_ *+ p*_2_ instead of two packets, and broadcasts it to nodes 1 and 3. Encoding operation in node 2 is possible if the destination nodes (1, 3) are able to extract the original packets. To show that decoding is possible in node 3, let us assume that encoding is performed in node 2 for the transmissions that are directed to node 1 and 3. Original packets vector is then composed of two packets *P*_1×2_ = [*p*_1_, *p*_2_]. Let us also select the following static coefficients in the matrix *V*_2×2_:

(1) $$V_{2\times 2}=\left[\begin{array}{cc} v_{11} & v_{12}\\ v_{21} & v_{21} \end{array}\right]=\left[\begin{array}{cc} 1 & 1\\ 0 & 1 \end{array}\right].$$

The vector of encoded packets is calculated, as follows:

(2) $$C_{1\times 2}^{T}=V_{2\times 2}\times P_{1\times 2}^{T},$$

(3) $$C_{1\times 2}^{T}=\left[\begin{array}{c} c_{1}\\ c_{2} \end{array}\right]=\left[\begin{array}{cc} 1 & 1\\ 0 & 1 \end{array}\right]\times \left[\begin{array}{c} p_{1}\\ p_{2} \end{array}\right]=\left[\begin{array}{c} p_{1}+p_{2}\\ p_{2} \end{array}\right],$$

where $P_{1\times 2}^{T}$ is a transpose vector of $P_{1\times 2}^{}$. Thus, two encoded packets should be received by node 3 to perform the decoding. The packet *c*_1_ *= p*_1_ *+ p*_2_ is constructed in node 2 and *c*_2_ = *p*_2_ must be buffered in node 3. Since the packets are a series of bits (values from the field GF(2)), the XOR bitwise operator is used (*p*_1_ *+ p*_2_*= p*_1_⊕*p*_2_). The static coding vectors are not attached to the encoded packets, but they should be known in a decoding node.

Node 3 can decode the original packets by performing the following operations

(4) $$P_{1\times 2}^{T}=V_{2\times 2}^{-1}\times C_{1\times 2}^{T},$$

(5) $$V_{2\times 2}^{-1}={\left[\begin{array}{cc} 1 & 1\\ 0 & 1 \end{array}\right]}^{-1}=\;\left[\begin{array}{cc} 1 & -1\\ 0 & 1 \end{array}\right],$$

(6) $$P_{1\times 2}^{T}=\left[\begin{array}{c} p_{1}\\ p_{2} \end{array}\right]=\left[\begin{array}{cc} 1 & -1\\ 0 & 1 \end{array}\right]\times \left[\begin{array}{c} c_{1}\\ c_{2} \end{array}\right]=\left[\begin{array}{c} c_{1}-c_{2}\\ c_{2} \end{array}\right].$$

The following equation is valid in the field GF(2):

(7) $$c_{1}-c_{2}=c_{1}⊕c_{2}.$$

Thus, in node 3, the packet *p*_1_ is extracted by the calculation *p*_1_ *= c*_1_ − *c*_2_ *= c*_1_⊕*c*_2_ (bitwise XOR operation), and the packet *p*_2_ = *c*_2_ is buffered in node 3.

The broadcast nature of wireless networks becomes a natural NC opportunity, since nodes can simultaneously receive broadcasted transmissions. Nodes that perform an encoding operation have to know which packets can be encoded (depending on the decoding possibility of the nodes that will receive coded packet). Even more, each node should also remember the packets that have already been sent, in order to use them for potential decoding. Also important is that inter-session network coding operations have to be performed in intermediate nodes. 

We expect that, while using the NC in the 6LoWPAN-based network, we can decrease the overall traffic volume that is generated by the WSN’s nodes, what leads to the decrease in the energy consumption in the whole network.

### 3.2. IPv6 over Low-Power Wireless Personal Area Networks (6LoWPAN)

In [36], the 6LoWPAN RFC4944 standard was introduced to support IPv6 packets transmission in the IEEE802.15.4-based sensor networks. The Maximum Transmission Unit (MTU) of the IPv6 streams cannot be smaller than 1280B, while the IEEE 802.15.4 standard offers a maximum frame payload of 127B [37]. The 6LoWPAN provides an adaptation layer that is located between the MAC layer and the IPv6 protocols stack. Additionally, it ensures IPv6 packets fragmentation to cope with small payloads. Besides, it allows for deep headers compression, in order to minimise the upper layers overhead. Since the IEEE802.15.4 standard offers a maximum 250 kbps data rate, the IPv6 protocols stack overhead would significantly reduce the network goodput. To increase sensor nodes resources utilization, the 6LoWPAN proposes—as an option—the so-called route-over routing mechanism. Fragments of IPv6 packets are transmitted to the destination nodes without reassembling at intermediate nodes, and without forwarding the full IPv6 packets at the network layer. This overcomes the problem of a larger memory size that is required in the nodes to collect fragments before reassembling. Unfortunately, in lossy sensor network, the route-over routing increases the packet loss ratio. Therefore, the traditional forwarding method (mesh-under routing) is often applied in the software used in sensor motes (i.e., in the Contiki Operating System).

### 3.3. Network Coding for 6LoWPAN (NC6LoWPAN) WSNs

As has already been mentioned, the main goal of the NC6LoWPAN implementation in 6LoWPAN-based WSNs is to decrease the overall network load, even if the network is not overloaded, which is what leads to saving the energy consumed by sensor motes. The following three basic assumptions were taken by the authors of this paper:

1. NC6LoWPAN should be applied for unicast transmission. Most of the sensor networks are deployed to transfer unicast streams from end sensors to a border gateway (and further to the Internet or cloud) or/and from the decision center to the actuators (associated with the sensor motes).
2. This technique should be easily implemented in sensor motes, possibly without any modifications in the MAC layer drivers.
3. It should be optimized to minimize node’s resources utilization, i.e., it should be equipped with the operations tailored for sensor network to minimize node’s memory usage, minimize additional signaling required for encoded packets acknowledgments, and decrease the overall traffic in the WSN (what should lead to the energy consumption decrease).

**Figure 2:**
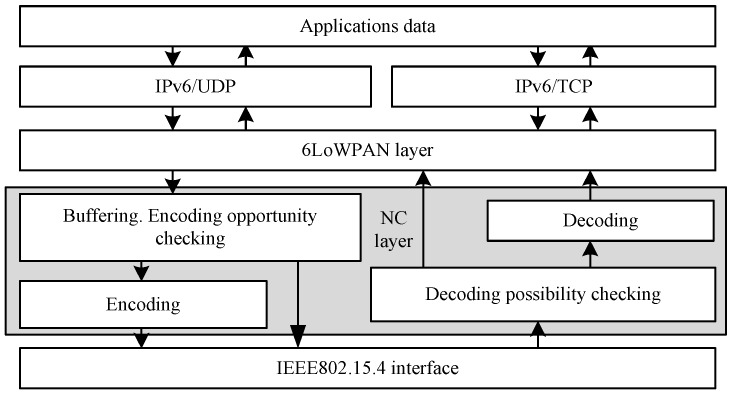
Location of the Network Coding (NC) layer.

In the NC6LoWPAN, the encoding and decoding operations are performed in the NC layer at the bottom of the 6LoWPAN layer, as it is shown in Figure 2. Such location of the NC entity avoids the modification of the MAC driver software, but it requires 6LoWAPN packets buffering in the NC layer, in order to ensure coding opportunity. Most of the NC techniques proposed in the literature (i.e., in [13]) assume that the native packets are not delayed for performing coding operation, but only buffered waiting for wireless medium access. Such assumption practically limits the network coding utilization to highly loaded or congested nodes. Thus, some authors proposed (for example, in [27,38]) to intentionally delay the packets in intermediate nodes of the IEEE 802.11-based network, allowing for increasing the coding opportunity. This idea is also used in the NC6LoWPAN, since the sensor network’s traffic can often be at low level, what could limit the coding efficiency. Appling additional packet delays, we should remember that it can influence the upper layer protocols (i.e., Transmission Control Protocol), which are packet delay and packet reordering sensitive. The NC6LoWPAN was assumed to be used with the User Datagram Protocol (UDP) units. This assumption seems to be reasonable for WSNs, since more services that were handled in the WSN are based on UDP transmissions. Besides, the communication techniques dedicated for wideband wireless networks allow for collecting all overheard packets and then use them during encoding and decoding operations. In addition, Katti et al., as showed in [13] that many network coding implementations work well in IEEE 802.11-based networks, where the node’s memory resources are not significantly restricted, while the 6LoWPAN-based WSNs are mainly composed of nodes with many specific functions and limitations. One of the restrictions of the NC application in such networks is the IPv6 headers compression, where some of the header fields can be changed from hop-to-hop. Additionally, we cannot occupy too much memory for NC buffers since the 6LoWPAN allows for packets’ fragmentation, which is what requires that nodes have to reserve the memory for merging fragments. Otherwise, the native datagrams (fragments) will be lost. All of these problems need some effort, mainly by the reduction of the NC signaling overhead, reduction of the coding/decoding range (one-hop), excluding the broadcast and multicast transmissions from coding operations, and other described in this paper.

In order to increase the coding opportunity in softly loaded WSNs, the native packets have to be buffered and slightly delayed in some nodes. What is more, the NC6LoWPAN does not collect all of the overheard packets, because of the sensors’ memory limitations. Thus, the main function of the NC layer in the NC6LoWPAN is to buffer the native unicast packets belonging to different sessions, check the coding opportunity, prepare encoded broadcast packets using bitwise XOR operation (according to Equation (7)), and perform decoding. The location of the NC layer that is shown in Figure 2 provides implementation simplicity, which is required in resource-limited sensor nodes. Furthermore, the NC layer allows for coding and decoding of the 6LoWPAN datagrams rather than the IPv6 packets.

**Figure 3:**
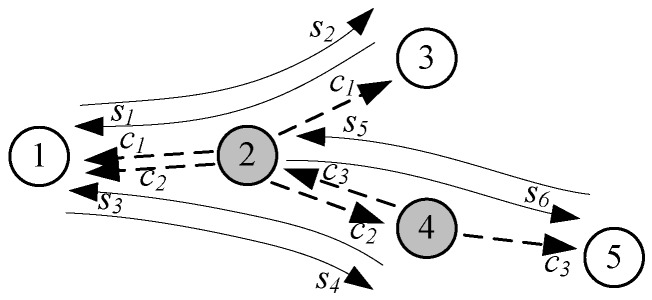
Network coding opportunity in intermediate nodes.

The encoding is performed in the intermediate nodes using native unicast packets, which belong to different sessions. As has already been mentioned, to increase network coding opportunity during normal WSN operation (at low traffic volume), these packets have to be buffered in the intermediate nodes. The size of buffers is limited by the node’s memory that has been allocated to the NC operations. The encoded packet can be formed if at least two native packets transferred to different next hop nodes are buffered. Figure 3 shows six IPv6 unicast streams (*s_1_*...*s*_6_). Only some packets crossing nodes 2 and 4 can be encoded. For example, packets from the stream *s*_1_ and *s*_2_ can be encoded only if they meet in node 2. Broadcast encoded stream (MAC layer broadcast) is depicted as *c*_1_. Similarly, packets from the streams *s*_3_ and *s*_4_ can be encoded in node 2, while packets from the streams *s*_5_ and *s*_6_ can be encoded in node 4.

The proposed network coding mechanism decreases the overall traffic in a sensor network (particularly in synchronised network). However, it should be noticed that native packets that are buffered in the intermediate nodes increase the overall end-to-end packets delay. The NC techniques often detect poor links (for example, based on the expected transmission count metric) to limit coding operation and then to decrease the number of decoding errors [13]. At this stage, to keep the NC6LoWPAN simplicity, it was assumed that sensors do not detect poor links. Potential errors during transmission of encoded packets affect decoding errors. Many solutions for the IEEE 802.11-based networks provide some kind of error correction mechanisms allowing for acknowledging the correctly received encoded packets (ACKs and pseudo-broadcast transmission in [13]). Similar mechanisms can also be used in WSNs, but they introduce additional signalling traffic, especially unwanted in a sensor network. It was assumed that the higher packet loss ratio resulting from decoding errors could be accepted from many WSN services point of view if the network lifetime will be extended. Nevertheless, to limit such effect, the next version of the NC6LoWPAN will be equipped with a relatively simple solution allowing for links quality detection. Additionally, to avoid instability in network operation that is caused by potential decoding errors, the signalling packets are not buffered and encoded (e.g., multicast Neighbour Discovery or RPL packets). The NC buffering time directly influences the coding efficiency and the network operation, which is discussed in Section 4.

The encoding procedure at the NC layer is described in Algorithm 1. Native 6LoWPAN packets, as received from the upper layer (named *CurrentNativePackets*), are checked according to message type (unicast or multicast, user data or signalling messages) in Steps 2–14. The current native packets that are excluded from further coding process are sent directly to the MAC layer (Step 8, 13), and then to the next hop node. Remaining current native packets can potentially be encoded depending on the node’s role recognised in Steps 15–20.

In the source nodes (*CurrentNodeRole* = *SourceNode*), the current native packets are sent to the MAC layer (Step 24) without encoding (decoding is not possible) if they are sent directly to the neighbouring sink node (checked in Step 23). If the current native packet is sent to the destination node via a neighbouring node (Step 24), then it has to be copied (Step 26). The copy is stored in the NC buffer (Step 30), while the current native packet is sent immediately to the MAC layer (Step 31). The copy of the current native packet (named *BufferedNativePacket*) can be used later on in case of a decoding necessity (the encoded packets received in the future can contain native packets retained in the NC buffer). Otherwise, the buffered native packet will be deleted from the NC buffer after the timeout *tz*. The value of *tz* directly influences the decoding possibility. By selecting a too low value of *tz*, the encoded packets cannot be decoded correctly in the node because of lack of the buffered native packets in the NC buffer, and in consequence, the coding gain will be decreased. A too high value of *tz* engages the node’s memory resources to keep native packets that cannot be used for decoding. To control appropriate timers reacting to timeout *tz*, current system time is written in the *BufferedNativePacket_tp* variable during the current native packet buffering (Step 28). Moreover, *BufferedNativePacket* is marked by the *BufferedNativePacket_role* variable, indicating that the current node is a source of the packet (Step 27). 

**Algorithm 1.** Encoding Procedure at the NC Layer**Input:***CurrentNativePacket* received from 6LoWPAN layer, *tc*—current system time.**Output:***OutputPacket* prepared for sending to MAC layer.1: Initialize: *NCOpportunity =* FALSE2: Read *DestAddr* of the *CurrentNativePacket*3: **if**
*DestAddr* = *UnicastAddr*
**then**4:  Read *NextHeader* field of the *CurrentNativePacket*5:  **if**
*NextHeader* = *ICMPv6*
**then**6:   Read *MessageType* of the *CurrentNativePacket*7:   **if**
*MessageType* = *RPL* or *MessageType* = *IPv6NeighborDiscoveryTypes*
**then**8:    *OutputPacket* ← *CurrentNativePacket*9:   **end if**10:  **end if**11: **end if**12: **if**
*DestAddr* = *MulticastAddr*
**then**13:  *OutputPacket* ← *CurrentNativePacket*14: **end if**15: Read *SrcAddr* of the *CurrentNativePacket*16: **if**
*SrcAddr* = *CurrentNodeAddr*
**then**17:  *CurrentNodeRole* ← *SourceNode*18: **else**
19:  *CurrentNodeRole* ← *RelayNode*20: **end if**21: **if**
*CurrentNodeRole* = *SourceNode*
**then**
22:  Read *NextHopAddr* from the forwarding table of the current node23:  **if**
*NextHopAddr* = *DestAddr*
**then**24:   *OutputPacket* ← *CurrentNativePacket*25:  **else**26:   *BufferedNativePacket* ← Copy of *CurrentNativePacket*27:   *BufferedNativePacket_role* = *SourceNode*
28:   Set *BufferedNativePacket_tz* = *tc*29:   *NCBuffer* ← *BufferedNativePacket*30:   *OutputPacket* ← *CurrentNativePacket*31:  **end if**32: **end if**33: **if**
*CurrentNodeRole* = *RelayNode*
**then**34:  **for each**
*BufferedNativePacket* ∊ *NCBuffer*
**do**35:   *NCOpportunity* ← Check NC opportunity (*BufferedNativePacket, CurrentNativePacket*)36:   **if**
*NCOpportunity* = TRUE **then**37:    *NetworkCodedPacket* ← *Encode* (*BufferedNativePacket, CurrentNativePacket*)38:    *OutputPacket* ← *NetworkCodedPacket*39:   **else**
40:    *BufferedNativePacket* ← *CurrentNativePacket*41:    *BufferedNativePacket_role* = *RelayNode*42:    Set *BufferedNativePacket_tp* = *tc*
43:    Set *BufferedNativePacket_tz* = −144:    *NCBuffer* ← *BufferedNativePacket*
45:   **end if**46:  **end for**47: **end if**

**Figure 4:**
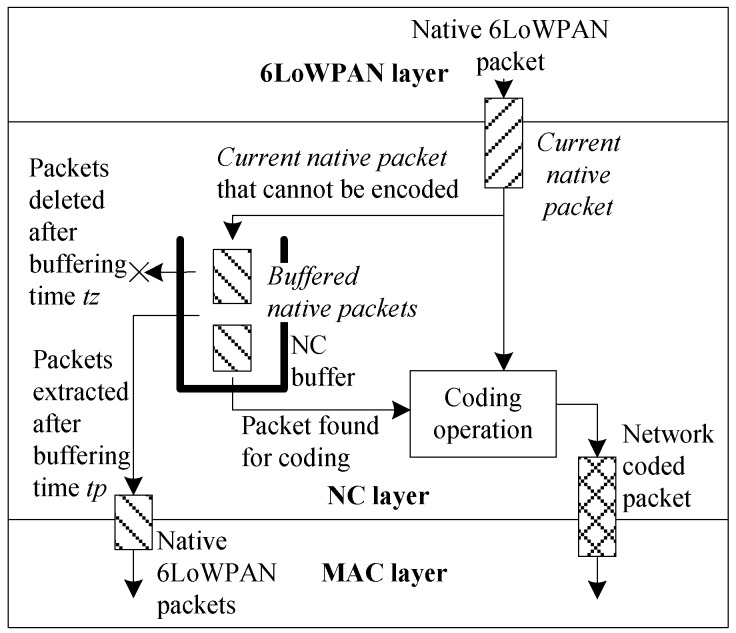
Network coding scheme in intermediate nodes.

**Figure 5:**
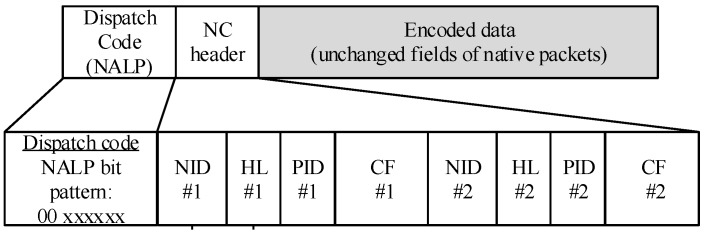
NC header format (for two encoded packets).

A network coding procedure in an intermediate node (*CurrentNodeRole* = *RelayNode*) starts in Step 33. Additionally, Figure 4 graphically illustrates coding scheme. The NC buffer is searched (Step 34) for buffered native packets that can be used for coding operation (Step 34). The buffered native packets meeting the coding rules are extracted from the NC buffer and are coded with the current native packet (Step 37). The coding rule requires that the buffered native packets, which are stored in the NC buffer, will be sent to their final destination over the node from which the current native packet was received. The encoded packet (Step 38) preceded by a Dispatch and NC layer headers (Figure 5) is sent to the MAC layer and broadcast. In order to simplify the decoding procedure, the identifiers of the recipients (hash of the recipients’ MAC addresses) are added to the NC header. 

If the coding operation cannot be performed, the current native packet is added in Step 44 to the NC buffer for a short time (*tp*) to give a chance for future encoding (it becomes buffered native packet). To control timeout *tp*, the current system time is written in the *BufferedNativePacket_tz* variable during the current native packet buffering. The value of tp is a parameter that directly influences the coding efficiency, but also the end-to-end packets delay. This value should be carefully selected, since an additional delay cannot be acceptable for some services. Buffered native packets that have not been used in coding operation are extracted from the NC buffer, sent to the MAC, and then transmitted to its next hop node. In the meantime, the copy of this packet is still buffered for the time *tz* for further possible decoding process. To do so, *BufferedNativePacket_tz* variable is used in Step 43 (value −1 is set, to indicate that buffered packet is ready for network coding). Both the time and space complexity of Algorithm 1 is *O*(*n*), and it directly depends on the NC buffer length*.*

Algorithm 2 illustrates a timers control procedure. Buffered native packets that have not been used for coding or decoding operations are deleted after time *tz* in source nodes (Step 4) and after time *tp + tz*, in relay nodes (Step 16). 

**Algorithm 2.** NC Buffer Timers Control**Input:***NCBuffer, tc*—current system time, *tz*, *tp*.**Output:***OutputPacket* prepared for sending to MAC layer.1: **for each**
*BufferedNativePacket* ∊ *NCBuffer*
**do**2:  **if**
*BufferedNativePacket_role* = *SourceNode*
**then**
3:   **if** (*tc* − *BufferedNativePacket_tz*) ≥ *tz*
**then**4:    Delete *BufferedNativePacket*
5:   **end if**6:  **end if**7:  **if**
*BufferedNativePacket_role* = *RelayNode*
**then**
8:   **if**
*BufferedNativePacket_tz* < 0 **then**9:    **if** (*tc* − *BufferedNativePacket_tp*) ≥ *tp*
**then**10:     *OutputPacket* ← copy of *BufferedNativePacket*11:     *BufferedNativePacket_tp* = −112:     *BufferedNativePacket_tz* = *tz*13:    **end if**14:   **else**15:    **if** (*tc* − *BufferedNativePacket_tz*) ≥ *tz*
**then**16:     Delete *BufferedNativePacket*
17:    **end if**18:   **end if**19:  **end if**20: **end for**

The decoding operation can be performed in each node, but to simplify, the implementation of the mechanism it is executed in the next hop nodes (nodes that are the neighbours of the coding nodes). The decoding procedure is described in Algorithm 3. 

The NC layer distinguishes encoded packets from native packets in Step 2 based on the dispatch code attached at the beginning of each packet (Figure 5). As proposed in RFC4944, the NLAP (Not a LoWPAN) bit pattern is selected to be sent with encoded data (Step 3). The original types of received packets are mapped on the 6-bits xxxxxx pattern. The native packets are directly sent to the 6LoWPAN layer in Step 25. Besides the dispatch header, the NLAP packets (encoded) are composed of the NC layer header and encoded data field. Only unchanged fields of the native 6LoWPAN packets can be used to prepare encoded data. The remaining ones have to be attached to the NC header (some 6LoWPAN fields are changing from hop-to-hop). The structure of the NC header, in the case of mixing of two native packets (#1 and #2), is shown in Figure 5. It contains identifiers of the nodes (hash of the MAC addresses) that are the recipients of the packet (*NID*#1 and *NID*#2), 6LoWPAN header fields that are changed from hop-to-hop (*CF*#1, *CF*#2), lengths of the *CF* fields (*HL*#1, *HL*#2), and native packets identifiers (*PID*#1, *PID*#2) that are required for separation of the packets from different streams. Encoding of compressed and frequently changing headers causes the NC header size to be relatively high. This limits the number of native packets that can be mixed together, and finally leads to the smaller value of coding gain (percentage of encoded packets versus all native packets that are generated in the network). The encoded shortest packets are padded by zeros to match the largest packet. The length of the packets can be found in the IPv6 compressed headers (payload length) or in the 6LoWPAN fragment header (datagram size).

To process encoded packets, the nodes compare its *CurrentNodeID* with *NID*s that are attached to the NC header (Steps 4–8). If the recipient finds in Steps 10–15 buffered packet that can be used for decoding, the decoding operation will be performed in Step 17 (*BufferedNativePacket* is XORed with *ReceivedPacket*). Otherwise, the decoding operation is not performed. This leads to decoding errors, and in consequence, to packets losses (Step 19). Decoding errors can be decreased by selecting a higher value of *tp*. On the other hand, a higher value of *tp* leads to ineffective utilisation of the sensor nodes recourses. The NC6LoWPAN does not acknowledge decoding errors. It accepts more errors, but it needs smaller memory size to perform network coding operations and increases sensors lifetime. Similarly to Algorithm 1, the time and space complexity of Algorithm 3 is also *O*(*n*) and it depends on the NC buffer length.

**Algorithm 3.** Decoding Procedure at the NC Layer**Input:***ReceivedPacket*—packet received from MAC layer (encoded or native 6LoWPAN packet), *CurrentNodeID.***Output:***CurrentNativePacket* prepared for sending to 6LoWPAN layer.1: Initialize: *Decode, Matched* = FALSE2: Read *DispatchCode* from the dispatch header of the *ReceivedPacket*3: **if**
*DispatchCode* = *NLAP*
**then**4:  **for**
*i* ← 1 to 2 **do**5:   **if**
*NID_i_* = *CurrentNodeID*
**then**6:    *Matched* ← TRUE7:   **end if**8:  **end for**9:  **if**
*Matched* = TRUE **then**10:   **for each**
*BufferedNativePacket* ∊ *NCBuffer*
**do**11:    *Decode* ← Check decoding possibility (*BufferedNativePacket, ReceivedPacket*) 12:    **if**
*Decode* = TRUE **then**13:     *BufferedNativePacket* ← Extract *BufferedNativePacket* from *NCBuffer*14:    **end if**15:   **end for**16:   **if**
*Decode* = TRUE **then**17:    *CurrentNativePacket* ← Perform decoding (*BufferedNativePacket, ReceivedPacket*)18:   **else**19:    Discard *ReceivedPacket*20:   **end if**21:  **else**22:   Discard *ReceivedPacket*23:  **end if**24: **else**25:  *CurrentNativePacket* ← *ReceivedPacket*26: **end if**

The algorithms presented in this section point that many mutually connected factors can influence the 6LoWPAN-based WSN efficiency. For example, by increasing the NC buffer length, we can expect higher NC opportunity, lower value of network traffic, and decoding errors, but the sensors need more memory resources and higher computing capability. Additionally, through increasing the NC buffer length, more packet can be delayed. Thus, reasonable trade-off should be found between these factors. The goal of this paper is to confirm the applicability of the NC technique in a real 6LoWPAN-based WSN, which is discussed in Section 4. Nevertheless, founding optimal values of the parameters used in the algorithms depending on the assumed requirements and constraints (applications, nodes, and network resources) requires further simulation investigations. 

## 4. Performance of NC6LoWPAN Mechanism

The NC6LoWPAN mechanism, as presented in Section 3, was implemented in an open source Contiki Operating System (OS) [26] that incorporates micro IPv6 protocols stack (uIPv6). It was ported to a number of microcontroller architectures, including the Texas Instruments MSP430. The performance of network coding in WSN was tested on the 2.4 GHz IEEE 802.15.4, 8-bit Amtel motes, as well as on the 8-bit Tmote Sky motes. The 8-bit Amtel AVR motes were equipped with AT86RF230 radio transceiver with high gain PCB antenna and RISC-based ATmega1284P microcontroller (AVRRAVEN), and with AT86RF230 radio transceiver with miniature PCB antenna, and AT90USB1287 microcontroller (RZUSBSTICK). The AVRRAVEN mote incorporates 128 KB ISP flash memory with read-while-write capabilities, 4 KB EEPROM and 16 KB SRAM, while the RZUSBSTICK uses 128 KB ISP flash memory with read-while-write capabilities, 4 KB EEPROM and 8 KB SRAM. The Tmote Sky motes utilise 48 KB flash memory and 10 KB RAM.

**Figure 6:**
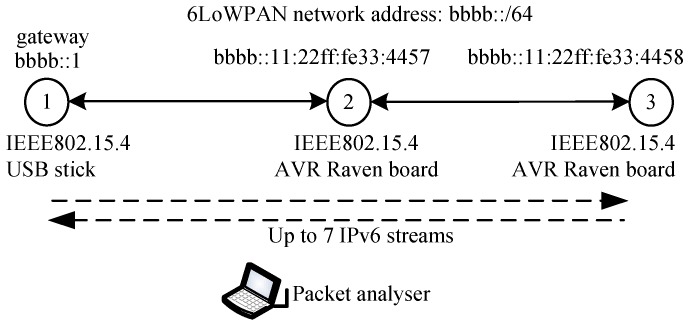
Simple two-hops Wireless Sensor Networks (WSN).

The NC6LoWPAN mechanism was examined in a testbedding environment. In the first scenario, the network was composed of three Amtel AVR motes (USB stick and AVR Raven boards) with Routing Protocol for Low power and Lossy Networks (RPL) [39]. The structure of the network is shown in Figure 6. Sensor nodes are located in such a way that propagation conditions do not bring packets loss due to jamming or signal attenuation. Thus, possible packet losses are caused by collisions or the sensors’ memory limitation (decoding errors). The packet analyser overhears all traffic and it helps packet end-to-end delay and packet loss ratio calculation. Such relatively simple scenario was selected to validate the correctness of NC6LoWPAN operations.

The traffic volume and its profile directly depend on the WSN application. If the sensors perform their standard role (just sensing), the data packet streams are mostly unidirectional (streams from sensors to border gateway). In such a case, there is no gain of the NC6LoWPAN usage (coding opportunity is low). It is more reasonable to use the NC6LoWPAN in WSNs with bidirectional traffic (Wireless Sensor & Actuator Network). The following characteristic of traffic exchanged between nodes 1 and 3 was assumed:

- Packets type: ICMPv6 (emulate bidirectional connectionless traffic, commonly used in sensor/actuator network).
- Packet size (ICMP data): 10 Bytes (constant, arbitrary selected).
- Number of streams: varying from 1 to 7 (to check an influence of traffic volume on correctness and efficiency of the NC6LoWPAN).
- Packets inter-arrival time distribution: uniform <0.4 s, 0.45 s> (arbitrary selected, which gives average 188 bps mean data rate).

To examine the sensor’s memory size allocation for the NC6LoWPAN, the NC buffer length was changed from 0 to 5 packets in each node, while 0 means that NC6LoWPAN is switched off, and 5, which means that up to 5 native packets can be buffered for coding or decoding operations. Thus, if higher NC buffer length is set up, then we can expect higher coding opportunity in a node (buffered native packets can be coded with other native packets which are currently received by a node). The native packets were buffered in the node for *tp* = *tz* = 0.5 s (values of *tp* and *tz* were arbitrary selected and have to be adjusted in the future depending on the applications end-to-end requirements). The overall traffic volume in the air (network load) was registered using a packet analyser capturing all of the IEEE802.15.4 frames that are generated in the network.

**Figure 7:**
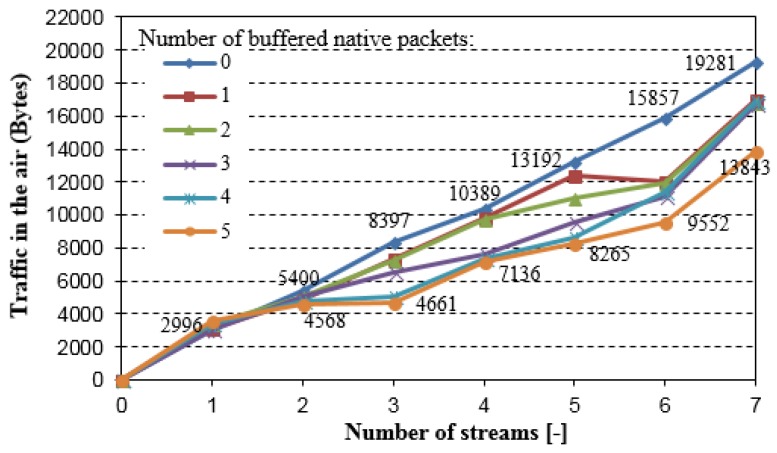
Overall traffic captured in the air (simple testing scenario)—number of buffered native packets = 0 means network without NC.

Figure 7 shows the overall traffic that is captured in the air as a function of the number of packet streams in one direction for different values of the NC buffer length (number of buffered native packets). If the NC buffer is full and a new native packet appears, the oldest packet stored in the NC buffer is immediately sent. The results confirm that the decrease in the overall network load directly depends on the traffic volume that is generated by the nodes, and on NC buffers capacity. It means that network coding in WSNs gives better results for higher traffic volume in the network. Unfortunately, the memory that is allocated for NC buffer is limited due to the sensor nodes resource limitations, which in consequence limits coding opportunity. Furthermore, the allocation of the memory for NC buffers in the nodes with significant memory limitation can increase the packets loss ratio because of the lack of free space for routed packets in the intermediate nodes.

**Figure 8:**
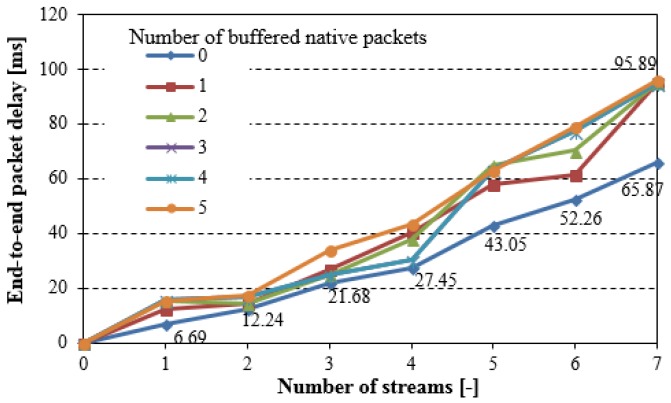
End-to-end packet delay (simple testing scenario).

The NC6LoWPAN implementation in the WSNs may cause additional end-to-end packet delay, as shown in Figure 8.

**Figure 9:**
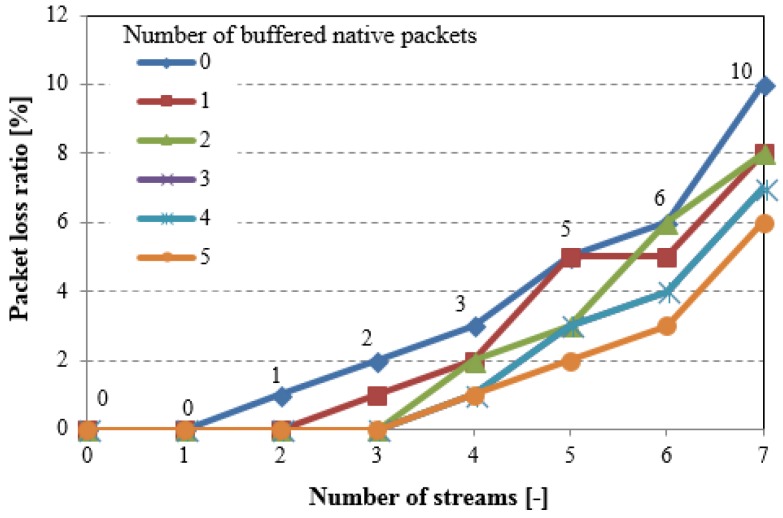
Packet loss ratio (simple testing scenario).

The number of buffered native packet does not significantly influence the packet end-to-end delay; however, it may decrease the packet loss ratio, as it is shown in Figure 9. This shows an impact of the nodes memory resources on the NC6LoWPAN efficiency.

**Figure 10:**
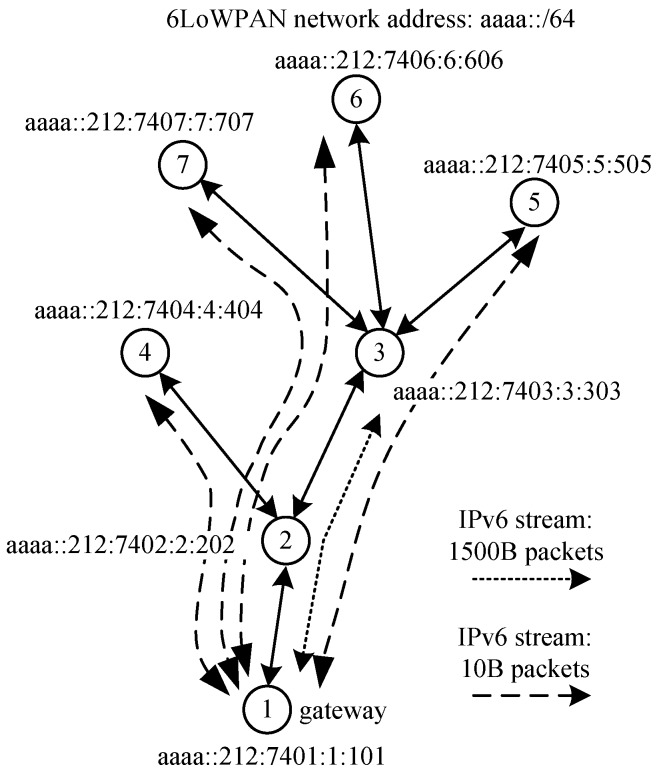
Multihop WSN.

The results presented in Figure 7, Figure 8 and Figure 9 are derived from a relatively simple sensor network. A more complex WSN scenario that is composed of seven Tmote Sky motes, as shown in Figure 10, was examined to validate these results. 

**Figure 11:**
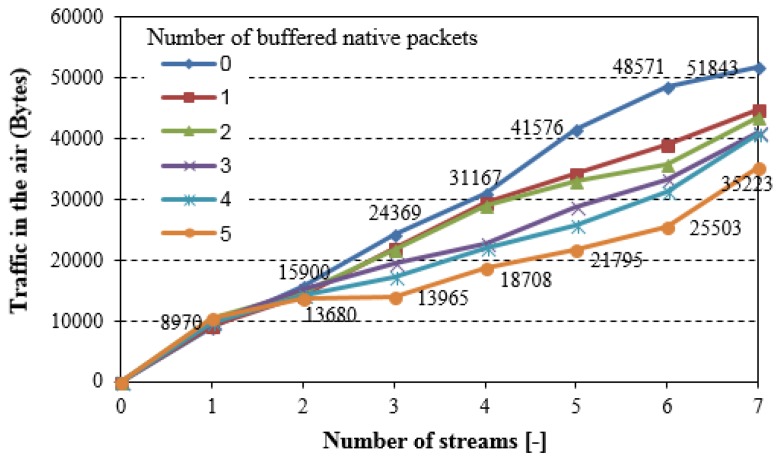
Overall traffic captured in the air (extended testing scenario).

Nodes from #3 up to #7 exchange IPv6 packets with the gateway (number of bidirectional packet streams varies from 1 to 7), with the same profile as in the simple scenario. Additionally, to demonstrate the influence of segmentation and reassembling processes that are given by the 6LoWPAN standard on the network performance, nodes #3 and #1 exchange 1500B IPv6 packets every 1 s. As a consequence, the overall traffic volume in the air is increased, as it is shown in Figure 11.

**Figure 12:**
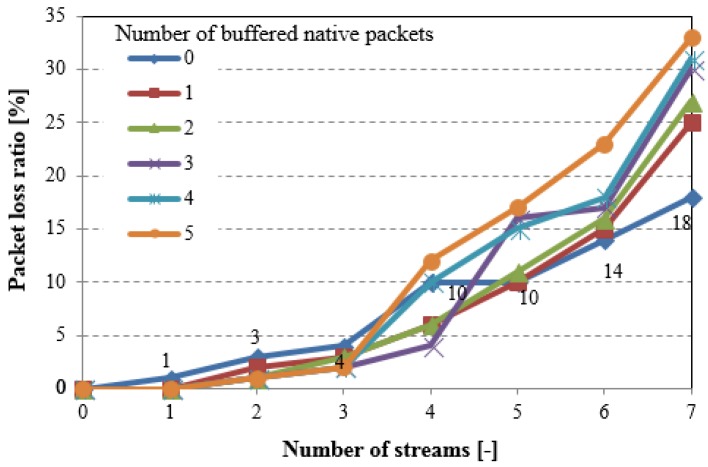
Packet lose ratio (extended testing scenario).

Both segmentation and reassembling actions require additional memory that is available in the nodes, which limits coding efficiency. The network coding operations reduce the overall traffic volume, but also they increase the packet loss ratio, as confirmed in Figure 12.

Many nodes are both source and relaying ones, so the lack of memory is a major reason of packets losses. This means that the proposed network coding technique can be used in sensor networks, but with moderate traffic level, and preferably in the nodes with relaying capabilities. We can decrease the packet loss ratio by limiting the number of packets that are stored in the NC buffer, but it also limits the NC efficiency through the overall traffic increase. Similar effect can be observed if the additional signalling will be provided to retransmit the lost packets. One of the method that can improve a packet loss ratio (keeping a traffic load decreased) can be the adaptive NC operations allowing for switch off the NC in the node or in a part of the WSN in the case of high traffic identified.

To show the NC6LoWPAN efficiency in terms of energy consumption, the overall energy that is consumed by the network were calculated. The energy consumed by the IEEE 802.15.4-based sensor motes that were used in the experiments were calculated according to the formula proposed in [40] for sent and received packets:

(8) $$E\left(send/receive\right)=m_{send/receive}\cdot size+b_{send/receive},$$

where, $m_{send/receive}$—the energy consumed by the sensor to send/receive 1B of the packet [J/B], $b_{send/receive}$—the energy consumed by the sensor to perform all of the operations to send/receive the data packet (transmission/reception initialization, the IEEE 802.15.4 header transmission/reception, the IEEE 802.15.4 acknowledge reception/transmission) [J], *size*—the size of the data packets to be transmitted [B].

**Figure 13:**
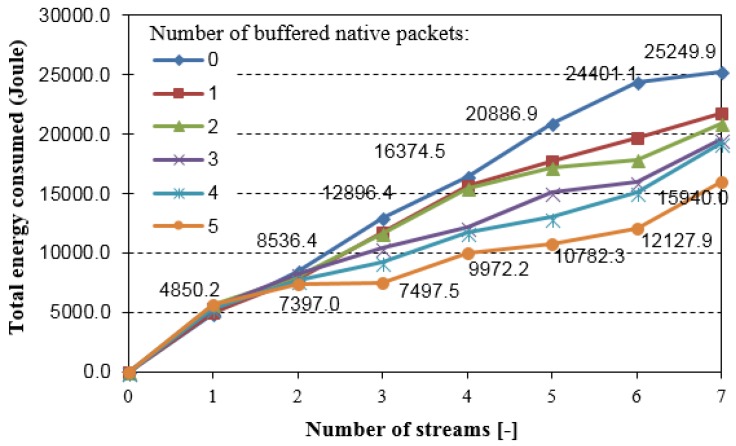
Total energy consumed in the network for packets transmission and reception (extended testing scenario).

Supporting by reference about the energy that is consumed by the Tmote Sky motes from [41], we can receive energy that is required for sending *n*-byte packets: *E*(*send*) = 0.12*n* + 3.54 [J], and for receiving *n*-byte packets: *E*(*receive*) = 0.12*n* + 4.03 [J]. By collecting all of the packets transmitted and received in the network presented in Figure 11 (with mean 28 Bytes: encoded and native data packets, including headers), we can assess the NC efficiency in terms of the total energy consumed in the network (*E*(*send*) + *E*(*receive*)). Figure 13 shows this assessment.

The highest energy consumption gain was observed when six streams were transmitted simultaneously in the network. Using the NC6LoWPAN, about 7% of the total energy was saved. It should be emphasized that the experiments were performed with a relatively high traffic volume generated during more than 1 hour, by each node of the networks (high total energy consumed). We can expect that the traffic will be much lower in many cases of the WSN. Thus, coding opportunities can also be decreased, and consequently, the energy consumption efficiency can be lower. Nevertheless, the results confirm that the NC6LoWPAN can be effectively used in the 6LoWPAN-based networks.

**Figure 14:**
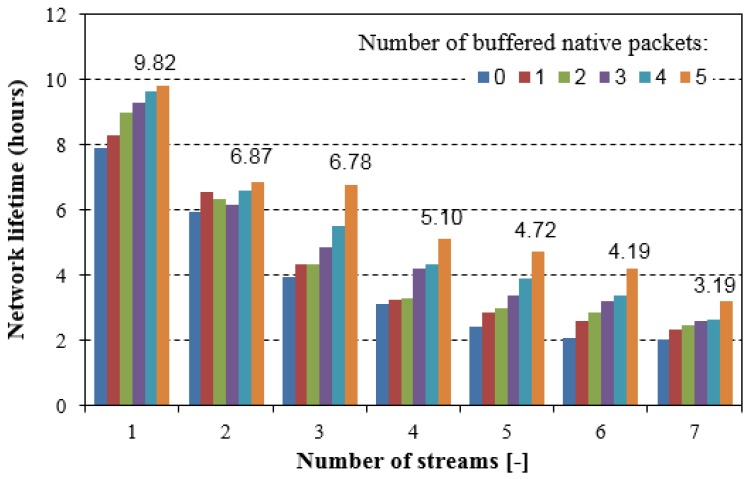
Estimated network lifetime (extended testing scenario).

Total energy consumption, as shown in Figure 13, is directly reflected in a WSN lifetime. Assuming that each node is powered by a battery with 1200 mAh capacity and 1.5 V nominal voltage, we have estimated the lifetime of the extended network shown in Figure 10. Thus, a node can use maximum energy equal to 6480 J. Since the experiments lasted for 67 min., we can linearly estimate the lifetime of the network that is composed of seven nodes. Figure 14 shows this estimation. 

A relatively low value of the network lifetime is a result of high traffic sent in the network during each experiment. In real WSNs, such traffic typically appears occasionally, thus the network lifetime is incomparably better. Nevertheless, an activation of the NC during higher traffic periods brings an increase in the lifetime. For example, for seven streams that were sent over the network, we can observe that estimated network lifetime is increased by about 60% if the NC is applied (with five native packets buffered in the NC buffers), when comparing to the network without the NC. Since the NC6LoWPAN mechanism is applied in real sensor motes and the results come from the experiments conducted in the real network, we mainly focused on the confirmation that the NC can bring benefits in overall traffic sent in the 6LoWPAN-based network, and in consequence, we can achieve lower overall energy consumption and a longer network lifetime. Moreover, the better benefits can be reached if the network is highly loaded. The NC6LoWPAN optimisation and its adaptation to specific applications used in the WSNs is left for further investigations. 

## 5. Conclusions and Future Work

The paper discusses the efficiency of the inter-session network coding technique in the IEEE802.15.4/6LoWPAN-based wireless sensor networks. The NC6LoWPAN mechanism was examined both in simple and more complex scenarios. The results confirm that the application of the network coding technique reduces both the overall traffic volume and the packet loss ratio, which leads to the sensor’s energy saving, but it may increase the end-to-end packet delay. The following recommendations should be considered for effective application of NC6LoWPAN in WSNs. Firstly, packets have to be buffered in the NC layer to provide a network coding opportunity, even in the softly loaded network. Secondly, an appropriate value of the buffering time has to be selected, depending on network type, traffic characteristics and user’s mobility. Thirdly, only unicast-based user data packets can be coded. 

The future work will be focused on the adaptive technique, allowing an increase in the coding efficiency in cognitive mobile sensor networks. The new solution should actively react on links quality, network structure changes due to nodes mobility, and the dynamic nature of wireless communications. It should also be independent of the broadcasted transmission and data link layer signaling protocols. Finally, it should incorporate some level of intelligence, allowing for us to adjust the network coding processes to a traffic type, routing information, and other possible cross layer information.
